# Equal division of parental care enhances nestling development in the Blackcap

**DOI:** 10.1371/journal.pone.0207757

**Published:** 2018-11-27

**Authors:** Konrad Leniowski, Ewa Węgrzyn

**Affiliations:** 1 Laboratory of Bioacoustics and Spectrophotometry, Faculty of Biotechnology, University of Rzeszów, Rzeszów, Poland; 2 Department of Zoology, Faculty of Biotechnology, University of Rzeszów, Rzeszów, Poland; Universidade de Sao Paulo Faculdade de Filosofia Ciencias e Letras de Ribeirao Preto, BRAZIL

## Abstract

Because parental care is costly, conflict between mates over their roles in reproduction seems unavoidable unless they both benefit from parental labour split equally between partners. In the current paper we analyse the division of parental investment in the Blackcap (*Sylvia atricapilla*), a species that experiences high nest predation. We show that both sexes invest in the incubation of eggs as well as feeding and brooding nestlings at a similar level. We also found that pairs which divided feeding duties more equally produced nestlings that grew faster. Faster nestling development enables earlier fledging in case of predation attempts at the end of nesting period. Thus parents who more evenly participate in provisioning may benefit from higher breeding success. Our findings suggest that in species under high risk of nest predation disparity in parental investment may not provide much benefit to parent’s residual reproductive value and that equality in parental duties constitutes a winning strategy.

## Introduction

Sexual conflict between parents may occur if the evolutionary interests of males and females do not coincide [1–3]. Cooperation of parents can enhance an individual’s inclusive fitness through increased offspring survival [4,5] but if care is costly offloading an unequal share of the work onto a mate may improve an individual’s survival and/or reproductive opportunities [6–9]. Despite the fact that both conflict and cooperation shape interrelationships between individuals, modern ecological theory mainly focuses on competition and diminishes the importance of positive interactions between organisms [10,11]. For example, monogamy with bi-parental care is the most common breeding pattern in birds [12], yet parental similarity has rarely been investigated so far. Most studies have tested the hypothesis of sexual conflict and focused on different investment strategies of males and females [1]. In contrast, the issue of how similar parents are in care behaviour has been studied less often.

Because parental care is costly [4,5] the conflict between mates over their roles in reproduction seems unavoidable unless they both benefit from parental labour split equally between partners. Care costs lead to conflict because parents value their own costs more than their partner’s costs. For there to be no conflict, parents must agree on the value of costs to each other. Thus the question arises as to how mates agree over how hard each of them should work and what environmental conditions may favour parental teamwork.

A number of factors may influence the strength of the conflict between males and females. Besides mating opportunities [6,13], the level of care required by nestlings is expected to strongly modulate parental conflict over care [14–17]. In species with slower nestling development, which requires less investments by parents per unit time, sexual conflict may be more pronounced because one of the parents is more capable of compensating for the shortfall of the other. Parents rearing fast-developing nestlings may both need to provide care at their full capacity to successfully raise their progeny. The brood-rearing environment is also expected to affect parental share of labour. Extreme environments require higher levels of parental care with both parent contributing fully. For example, avian eggs need to be kept within a narrow range of temperatures (36–38°C) for embryos to develop optimally [18,19]. Thus, in extremely low or high ambient temperatures incubation and brooding are shared approximately equally between the male and the female [20,21]. Also the risk of predation has been shown to influence parental care in birds [22–25]. Life-history theory predicts that high nest mortality may select for increased parental investment in incubation and feeding, to accelerate offspring development and minimize exposure to predators [16,26]. This hypothesis has gained considerable support so far [25,27–29].

In this paper, we analyse division of parental investment in the Blackcap (*Sylvia atricapilla*)*—*a species that experiences high nest predation and rapid development of nestlings [30]. Based on the data collected in a 6-year field study, we test the hypothesis that (i) parental duties are evenly distributed between pair members and (ii) equal division of labour between mates enhances nestling development. We also test whether faster growth increases nestling chance of survival in case of predation attempts at the end of nesting period.

## Methods

### Species and study area

The present investigation is a part of a larger project on behavioural ecology of the Blackcap conducted over the period 2008–2015. The study was conducted in the deciduous forest of the Fox Hill Reserve on the outskirts of Rzeszow, south-eastern Poland (DD: 50.009, 21.987).

The Blackcap is a small (about 13 cm long with a 7–8 cm wing length), migratory, open-nesting passerine that breeds throughout Europe. It prefers habitats characterized by dense tree and shrub vegetation [31]. It builds thin-walled, open cup nests in the shrub and herbaceous layers of forests [32]. The clutch size is three to six eggs laid on consecutive days. Incubation lasts about 12 days and nestlings stay in the nest for another 12 days, but they are able to leave the nest when 9 days old. Nestlings are typically fed and brooded by both parents: males develop brood patches similar to that of females (pers. observ., [33]). Brooding takes place throughout the nestling period but decreases prior to fledging when nestlings acquire endothermy [30]. All nestlings within a brood fledge at the same time ([30], pers. obs.). The Blackcap suffers from high rates of nest predation, mostly by rodents and corvids [34]. A number of studies demonstrated that on average only 30% of nests survive [30,35–39].

### Data collection

Nests were searched for by careful inspection of potential nest-sites after mapping male breeding territories in the spring. We searched for nests from mid-April until the end of June in all years of the study. We used the nest monitoring protocol described in [30]. Where possible we checked poorly concealed nests using binoculars to minimize the disturbance to incubating birds. We visited nests daily at random times of day (06:00 a.m.- 06:00 p.m) during the egg stage. Nests were visited on subsequent days until eggs hatched or a nest was depredated. During each visit we recorded the sex of an incubating parent to estimate the parental share of incubation. After hatching nests were visited daily between 06:00 p.m. and 08:00 p.m. to collect data on nest predation and nestling development. Last measurements were collected when nestlings were 8 days old as older nestlings may fledge when disturbed. In nests with nestlings older than 8 days we checked nest survival from the distance of about 2 meters to prevent early fledging caused by our presence. Such caution was absolutely sufficient as Blackcap nestlings do not leave their nest until approached very closely (about 30cm) or directly touched.

Altogether in years 2009–2014 we collected 934 observations of incubating parent from 106 nests. We also filmed 50 nests with nestlings aged 2–8 days. We used the micro-camera (Sony 1/4” CCD matrix, pinhole lens; Tokyo, Japan) connected to a laptop through a Pinnacle Studio 10 USB video converter. The micro-camera (of thumb-nail size) was placed about 25 cm from the nest and left for 1 hour to allow parents to resume their natural feeding activity. For more detailed description of camera mounting refer to [38]. After parents resumed their natural feeding behavior, each nest was filmed for 1 hour. All video recordings were made between 06.00 a.m. and 09.00 a.m. Each nest was filmed for 1 hour and the following data were later extracted from recordings: (i) male and female share in feedings, (ii) male and female share in brooding and (iii) male and female share of nest sanitation from parasitic larvae of blowflies (*Protocalliphora* sp). The latter two activities of each sex were analysed both in terms of frequency and duration (rounded off to the nearest whole second). Male and female identity was recognized based on sexual dimorphism between parents: a male has a black cap whereas a female has a brown cap. To minimize observer bias, blinded methods were used when all behavioral data were recorded and/or analyzed.

In 20 nests, at eight days of age, we both filmed parental activity and weighed nestlings before fledging, using a pesola scale (precision 0.1g). This data were used to test the effect of the level of parental parity on nestling development.

To test whether faster growing nestlings have higher chances of survival when forced to early fledge, we analysed the fate of broods that were naturally attacked by a predator at nine days of age. Blackcap nestlings typically stay in the nest for 12–13 days, but they are able to early fledge when nine days old if scared away by a predator. However, they not always can survive such early fledging (E. Węgrzyn, pers. observation). We wanted to know whether nestling mass is a predictor of survival in a case of fledging at the earliest possible time (i.e. when nestlings are 9 days old). During our project (2008–2015) we collected the data of nest predation and we found an undeniable attempt of predation on nine-day-old nestlings in up to three nests a year, which makes altogether 14 documented cases. We were sure that the analysed 14 nests were attacked by a predator because they were obviously torn and destroyed. We were able to assess nestling fate after a predator attack at nine days of age because all nestlings in our study area were banded. After fledging siblings stay together and parents feed begging nestlings in the proximity of the nest for 1–3 days before they disperse (E. Węgrzyn, pers. observation). So, it is possible to check whether the brood is alive or not and how many siblings survived a predator attack. Nestling survival was confirmed either by observation with binoculars or by mist-netting siblings. Because nest predation was monitored daily between 06:00 p. m. and 08:00 p. m., we searched for nestlings from destroyed nests on the following day. To find them we both carefully observed their natal territory and we listened to fledgling begging calls, which are quite audible once nestlings leave a nest. We considered that a nestling survived early fledging at the age of nine days if it was still alive on the subsequent day.

### Statistical analyses

All analyses were conducted using SPSS 20 software (SPSS Inc., Chicago, IL). The effect of parent sex on participation in incubation was tested using Linear Mixed Model with nest as a subject, incubation share as a dependent variable and sex of a parent as a fixed factor. The effect of parent sex on feeding rate was tested using Linear Mixed Model with nest as a subject, feeding rate as a dependent variable and sex of a parent and nestling age as fixed factors. Differences between males and females in brooding rate, brooding time, sanitation rate and sanitation time were analysed using a Wilcoxon Signed- Rank Test.

To test the effect of parental parity on nestling development first we calculated a parental parity coefficient (PC) as follows: PC = activity of the less investing parent / activity of the more investing parent. In such an approach, the more equal the division of work between parents, the closer the value of the coefficient is to 1. PC was calculated separately for three main parental activities: feeding (PCf), brooding (PCb) and sanitation (PCs) rate. Next, we tested the effect of parental parity on nestling development using General Linear Model with mean nestling mass in a nest before fledging (at day 8) as a dependent variable, PCf, PCb,PCs, feeding rate and an interaction between feeding rate and PCf as fixed factors.

To test the effect of nestling mass on the ability to survive a forced fledging at nine days of age caused by a predator attack we used a LMM with nest as a subject, siblings from a given nest as a repeated measure, nestling survival as a dependent variable and nestling mass as a fixed factor.

### Ethics statement

The study does not involve experiments on live vertebrates. All video recordings were made with a minimum disturbance to the nests and they did not cause the desertion of any nests or behavioural changes in parental activity. Additionally, to meet the requirements of good practice for wildlife research this study was carried out with approval of the Regional Management of the Environmental Protection in Rzeszów (the permit number: WPN.6205.30.2011.ŁL-2).

## Results

### Male and female participation in parental activities

**Fig 1:**
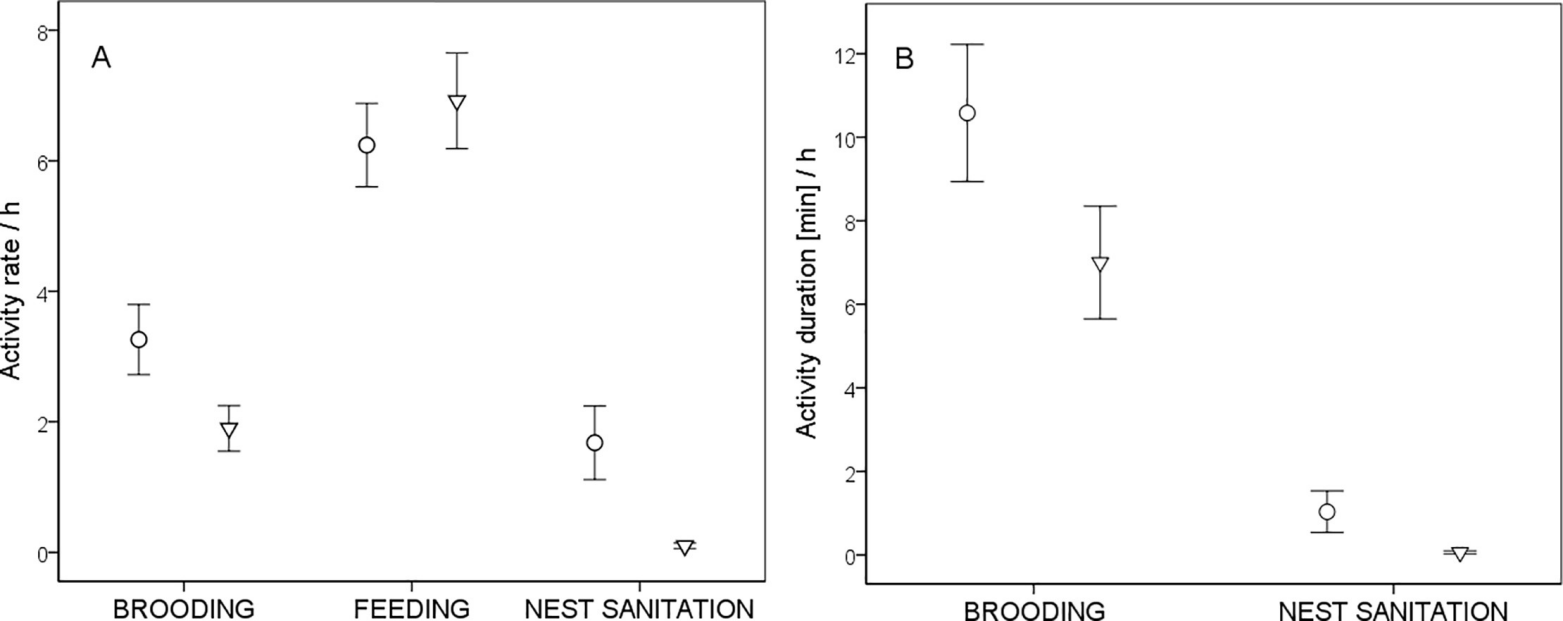
Blackcap male (▼) and female (●) parental investment during 1 hour observation, n = 50 nests. A–activity rate/h, B–activity duration/h. Bars show ± 1SE.

We analysed parental share in egg incubation, nestling feeding, nestling brooding and nest sanitation. Both parents participated in all listed activities (Fig 1). Males and females equally shared egg incubation (Table 1) and nestling provisioning (Table 1). Brooding and nest sanitation were female biased, both in terms of frequency and duration (Table 1). However, nest sanitation in Blackcaps does not constitute a considerable energetic expenditure–on average females spent about one minute per hour on cleaning activity (Table 1).

**Table 1:** Blackcap male and female participation in parental care.

	Egg incubation share		Feeding rate		Brooding rate		Brooding duration (s)		Nest sanitation rate		Nest sanitation duration (s)	
Sex	♀	♂	♀	♂	♀	♂	♀	♂	♀	♂	♀	♂
Mean	0.52	0.48	6.24	6.92	3.26	1.9	634	416	1.95	0.1	62	2.1
Min.	0	0	0	1	0	0	0	0	0	0	0	0
Max.	1	1	22	28	13	8	2160	1545	24	1	1406	45
SD	0.23	0.22	4.51	5.19	3.8	2.47	698.46	569.04	4.3	0.3	211.6	7.76
No. of nests	106	106	50	50	50	50	50	50	50	50	50	50
Difference	LMM: F = 1.520, p = 0.219		LMM: F = 1.269, p = 0.263		Wilcoxon test: Z = 4.196 **p < 0.001**		Wilcoxon test: Z = 4.996 **p <0.001**		Wilcoxon test: Z = 4.134 **p < 0.001**		Wilcoxon test: Z = 3.608 **p < 0.001**	

### The effect of the level of parental parity on nestling development

**Fig 2:**
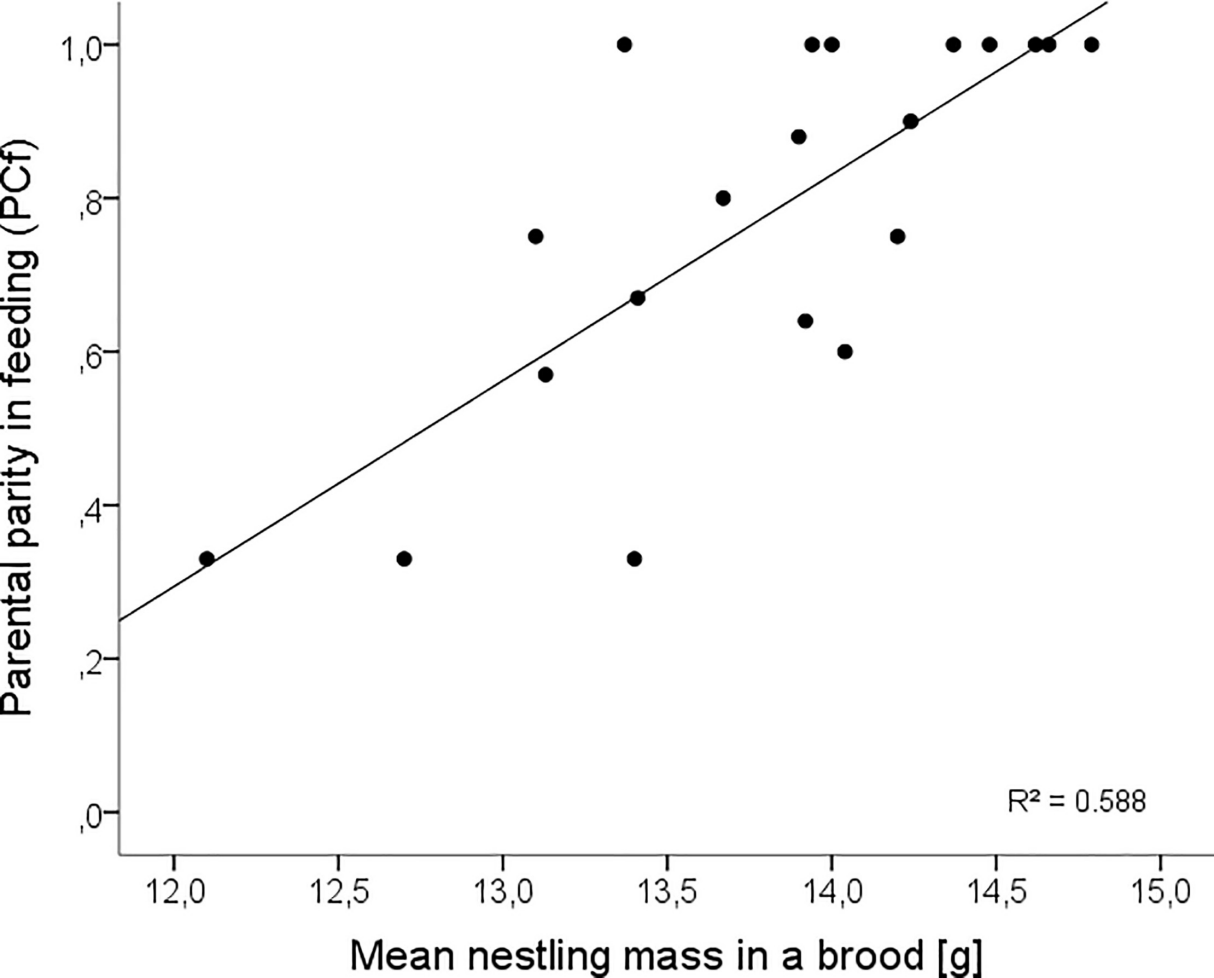
Relation between mean nestling mass in a brood prior to fledging, at eight days old, and the parental parity in feeding (PCf). The more equal division of work between parents the closer the value of PCf to 1. N = 20 nests.

The division of nest provisioning (PCf) had a significant effect on nestling development (Table 2). Nestlings whose parents shared feeding duties more equally grew faster than offspring of parents which had higher levels of disparity (Fig 2). The level of cooperation between parents in brooding (PCb) also significantly enhanced nestling growth but parental share in nest sanitation (PCs) had no effect on nestling development (Table 2). Feeding rate did not significantly differ between heavier and lighter broods, however nestling mass depended on the interaction between feeding rate and parental share in provisioning (Table 2). The model predicts that for every unit increase in feeding rate the effect of parental parity in feeding (PCf) on nestling mass is increased by 0.68. Although the effect of interaction is significant, it is quite subtle compared to the effect of the individual variable PCf.

**Table 2:** GLM on factors affecting nestling development and the estimate values of parameters.

Source	Exp (β)	F	Sig.
PCf	11.94	228.491	0.042
PCb	0.12	245.785	0.047
PCs	-0.12	34.947	0.119
Feeding rate	0.07	81.000	0.070
Feeding rate * PCf	0.68	171.259	0.049

Dependent variable: nestling mass

### The effect of nestling growth on the ability to survive an early fledging

**Fig 3:**
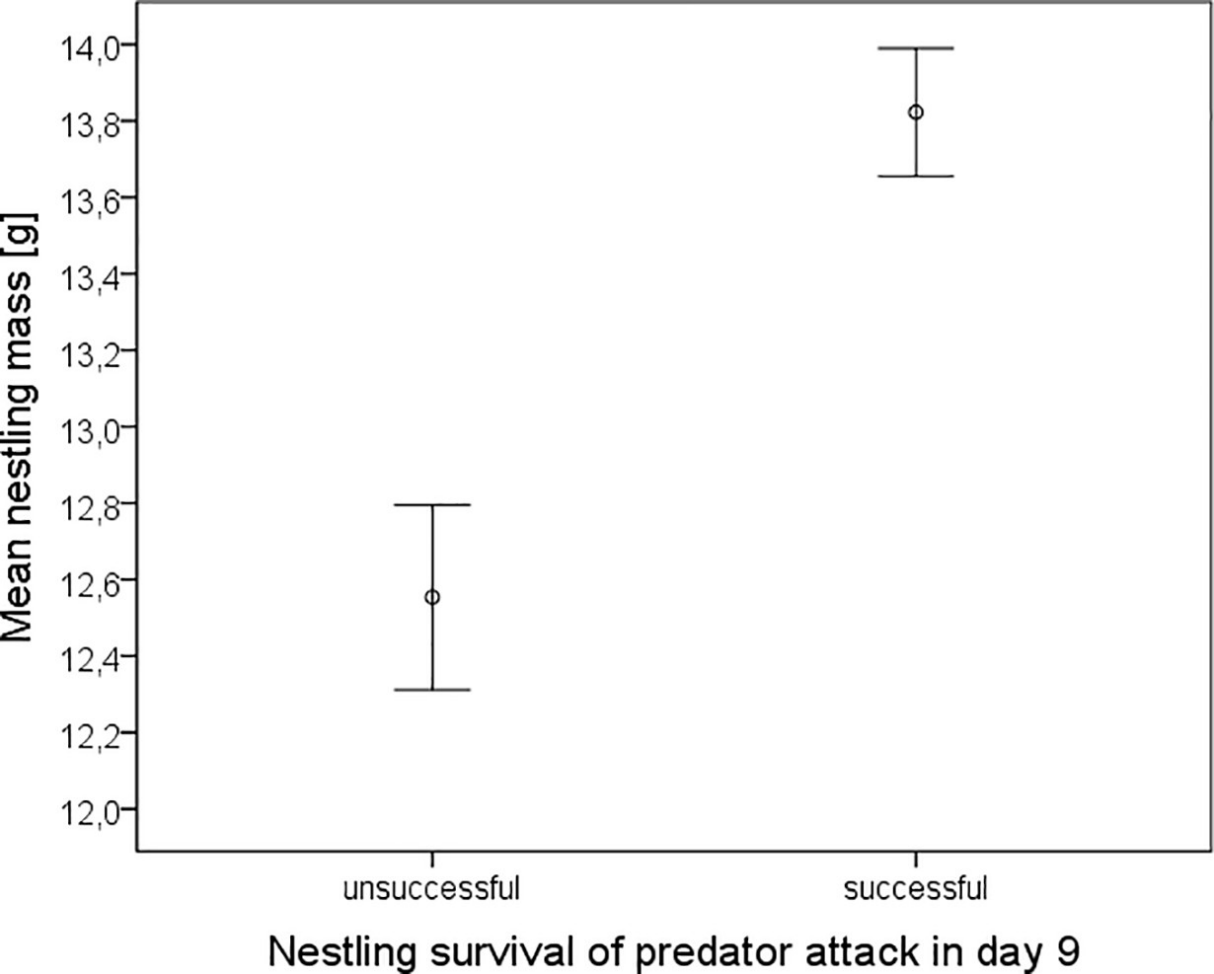
Relation between nestling mass and survival of nestlings forced to fledge at nine days of age because of a predator attack. N = 14 nests.

Of 14 broods forced to fledge at nine days of age because of a predator attack eight were confirmed to survive. Heavier nestlings survived better than lighter ones (Fig 3) and nestling mass had a significant effect on survival (F = 5.451, p < 0.001). Interestingly, three of eight successful broods survived partially. In these broods the lighter nestlings were missing.

## Discussion

Our study has demonstrated that in Blackcaps both sexes share main parental duties, namely egg incubation and feeding nestlings. We have also found that nestlings of parents that participated in feeding more equally gained higher mass before fledging even when offspring received no more visits in total. This is an important finding which shows that biparental care itself may not guarantee enhanced nestling development unless both parents equally share parental duties. Previous studies conducted by other researchers suggest that the division of parental care between mates plays an important role in nestling growth. It was experimentally demonstrated that the decrease of provisioning by one parent resulted in slower chick growth and poorer condition of fledglings in the European starling (*Sturnus vulgaris*) because compensation provided by the other parent was incomplete [40]. Similar findings were reported in Zebra finches (*Taeniopygia guttata*) [41]. Other studies revealed that the reduced investment by one parent leads to increased nestling starvation and mortality [42,43]. Our findings differ from the above cited studies in that equal division of parental care enhanced nestling growth irrespective of total feeding rate. Although feeding rate did not affect nestling mass significantly, we found a significant interaction between feeding rate and parental share in provisioning. The positive effect of parental parity on nestling growth increased with feeding rate, showing that the equal share of parental duties was more beneficial when both parents were diligent rather than lazy. Thus, despite of the occurrence of sexual conflict, which typically leads to unequal share of parental care between the sexes (reviewed in [44]), it seems that in certain circumstances, when fast nestling development is advantageous (for example under high risk of nest predation), disparity in parental investment may not provide much benefit to a parent’s residual reproductive value. We confirmed this assumption in our study, showing that parents who shared their duties more equally significantly enhanced the growth of their nestlings. Faster nestling development enables earlier fledging in case of predation attempt at the end of nesting period and thus parental parity may increase breeding success.

Interestingly, recent studies have revealed that in biparental species the reproductive success of a pair is at least partly affected by behavioural compatibility between mates [45–47] and this effect is independent of the genetic quality of parents [48,49]. Thus enhanced growth of Blackcap nestlings reared by more cooperative parents may be the result of better behavioural matches between breeding partners. Parents who divide care more equally may be more consistent or regular, which may make food delivery more efficient. Also, two parents feeding at the same rate may have more time to forage, and so deliver larger or better food items than one busy parent and one that reduces its effort.

Another factor that may favour equal investment by both sexes is the positive effect of provisioning on offspring recruitment [50]. It has been well documented that individuals with a higher body mass at fledging have higher survival rates than lighter ones [51–55]. Thus, diligent parents who share their duties with a partner may achieve multiple benefits that together outweigh advantages of saving energy for different purposes. On the other hand, bird parents may also employ strategies which prevent their mates from a reduced investment in offspring, which leads to more equal distribution of labour between pair members. For example, provisioning by one partner may be withheld until the other partner has provisioned, which results in a conditional cooperation in a form of alternation of feeding trips [56]. Such behaviour may be facilitated by parental synchrony in provisioning because it allows each parent to scrutinize the investment of its mate [57].

The most striking difference in the pattern of parental care between the Blackcap and some other studied species which exhibit bi-parental care is a high male involvement in all activities. In other species males typically do not participate in egg incubation [54], feed offspring at lower rates than females [23,58], but see [59] and spent much shorter time, if any, brooding nestlings [54]. We suggest that high male investment in parental duties in Blackcaps may result from nest predation pressure and the urge to shorten nestling exposure time to a predator.

The most female biased activity observed in our study was nest sanitation, which was also reported in other species [60,61]. However, cleaning the nest constituted a marginal amount in time budget of Blackcap females–on average about one minute per hour. Although parasitic load may significantly influence nestling condition and developmental rates in some species [62–65], this is not the case in the Blackcap [66]. Blackcaps build nests of lacy structure, which both prevents high infestations of ectoparasites and facilitates their effective removal by parents. As a result, the level of ectoparasitic load observed in Blackcap nests is low [66] and nest sanitation is not such a challenge in terms of time and energy as reported in other species suffering from high parasitic loads [61, 67]. Thus, despite the fact that most Blackcap males poorly participated in nest cleaning, the share of parental duties between sexes seems relatively equal in this species.

## Conclusions

In conclusion, in Blackcaps both sexes seem to pay similar costs and have similar benefits to caring. Parental parity improves an individual’s direct fitness which leads to equal involvement of both parents in rearing the brood. The main benefit of such strategy is a faster offspring development and a higher chance of survival during early fledging caused by predation attempts at the end of nesting period. This may be of crucial importance in a species suffering from heavy nest predation.
